# Metabolomics and Lipidomics Screening Reveal Reprogrammed Signaling Pathways toward Cancer Development in Non-Alcoholic Steatohepatitis

**DOI:** 10.3390/ijms24010210

**Published:** 2022-12-22

**Authors:** Eman A. Ahmed, Marwa O. El-Derany, Ali Mostafa Anwar, Essa M. Saied, Sameh Magdeldin

**Affiliations:** 1 Proteomics and Metabolomics Research Program, Department of Basic Research, Children’s Cancer Hospital 57357, Cairo 11441, Egypt; 2 Department of Pharmacology, Faculty of Veterinary Medicine, Suez Canal University, Ismailia 41522, Egypt; 3 Department of Biochemistry, Faculty of Pharmacy, Ain Shams University, Cairo 11566, Egypt; 4 Chemistry Department, Faculty of Science, Suez Canal University, Ismailia 41522, Egypt; 5 Institute for Chemistry, Humboldt Universität zu Berlin, Brook-Taylor-Str. 2, 12489 Berlin, Germany; 6 Department of Physiology, Faculty of Veterinary Medicine, Suez Canal University, Ismailia 41522, Egypt

**Keywords:** non-alcoholic steatohepatitis, hepatocellular carcinoma, metabolomic, lipidomic, pathway reprogramming

## Abstract

With the rising incidence of hepatocellular carcinoma (HCC) from non-alcoholic steatohepatitis (NASH), identifying new metabolic readouts that function in metabolic pathway perpetuation is still a demand. The study aimed to compare the metabolic signature between NASH and NASH-HCC patients to explore novel reprogrammed metabolic pathways that might modulate cancer progression in NASH patients. NASH and NASH-HCC patients were recruited and screened for metabolomics, and isotope-labeled lipidomics were targeted and profiled using the EXION-LCTM system equipped with a Triple-TOFTM 5600+ system. Results demonstrated significantly (*p* ≤ 0.05) higher levels of triacylglycerol, AFP, AST, and cancer antigen 19-9 in NASH-HCC than in NASH patients, while prothrombin time, platelet count, and total leukocyte count were decreased significantly (*p* ≤ 0.05). Serum metabolic profiling showed a panel of twenty metabolites with 10% FDR and *p* ≤ 0.05 in both targeted and non-targeted analysis that could segregate NASH-HCC from NASH patients. Pathway analysis revealed that the metabolites are implicated in the down-regulation of necroptosis, amino acid metabolism, and regulation of lipid metabolism by PPAR-α, biogenic amine synthesis, fatty acid metabolism, and the mTOR signaling pathway. Cholesterol metabolism, DNA repair, methylation pathway, bile acid, and salts metabolism were significantly upregulated in NASH-HCC compared to the NASH group. Metabolite–protein interactions network analysis clarified a set of well-known protein encoding genes that play crucial roles in cancer, including PEMT, IL4I1, BAAT, TAT, CDKAL1, NNMT, PNP, NOS1, and AHCYL. Taken together, reliable metabolite fingerprints are presented and illustrated in a detailed map for the most predominant reprogrammed metabolic pathways that target HCC development from NASH.

## 1. Introduction

A broad spectrum of liver manifestations translated from dysregulated metabolism is a characteristic feature of non-alcoholic fatty liver disease (NAFLD). The prevalence of NAFLD among the general population ranges from 11.2–37.2% [1]. Mechanistically, the disease is characterized by metabolic lipids upregulation within steatotic hepatocytes. Long-term steatosis can lead to hepatocellular ballooning, lobular inflammation, hepatocyte apoptosis, and non-alcoholic steatohepatitis (NASH) [2]. NASH is the most severe form of NAFLD, and it can be verified histologically in up to 47% of all NAFLD cases [3]. In previous cohorts, the prevalence of NAFLD-related HCC ranged from 41–49% of NAFLD without cirrhosis [4]. Hence, NASH became the second leading reason for HCC [4]. In two large studies in the Veterans Health Administration, 20% to 36% of non-cirrhotic NASH patients were diagnosed with HCC [5,6]. Furthermore, during analyzing the HCC cohort over the years, the researcher reported that 65% of the cases were raised on the NASH background without cirrhosis [7]. Despite being benign at early stages, NASH can aggressively progress into advanced fibrosis and cirrhosis, ending with hepatocellular carcinoma (HCC) [2,8]. With increased annual incidence, HCC arising from NASH background is now considered the fastest-growing disease [2,8]. However, it is usually diagnosed at the terminal stage of the disease because of the low sensitivity of the available screening tests [2,8]. 

The cellular metabolism is altered during carcinogenesis toward an adaptive hypoxic environment and evading apoptotic signals [9]. Growing evidence suggests that specific metabolites can exert a cancer-promoting effect through the modulation of diverse signaling pathways [10]. However, these metabolites are still elusive and incompletely understood [8,11]. Therefore, linking the metabolic reprogramming that hallmarks cancer within NASH-dysregulated metabolism might open the door to discovering novel metabolic readouts. In addition, it will aid in identifying possible biomarkers for the early detection of HCC in NASH patients [12]. However, alpha-fetoprotein (AFP) remains the primary tumor marker for HCC; its sensitivity and predictive values range from 25–65%. AFP levels are within the normal range in 40% of HCC patients. Additionally, only 20% of HCC patients have high levels of AFP in the early stages, while AFP levels can be high in HCC-free individuals. Accordingly, the American Association for the Study of Liver Diseases (AASLD) noted that it is unreliable for HCC screening [9]. 

Of note, increased dietary intake of fatty acids is associated with lipid metabolism imbalance in the liver [13]. Interestingly, dysregulation of fatty acid metabolism activates aberrant oncogenic signaling pathways involved in HCC development and progression [14]. Moreover, evidence highlights the upregulation of the tricarboxylic acid (TCA) cycle toward cancer progression. This alteration is thought to affect the activity of enzymes in glutamine metabolism, malate/aspartate, and citrate/pyruvate shuttles, which play a crucial role in cancer development and progression [15,16,17]. These disrupted metabolic pathways align with an impaired mitochondrial adaptation resulting in antioxidant activity impairment and ATP disruption, which is crucial in NASH to HCC transition [18]. Moreover, micronutrients such as vitamins are vital in maintaining physiological function and metabolism and impact disease progression [19]. Studies have focused on a specific vitamin; however, a broader outlook of the vitamins in NASH and NASH-HCC remains unexplored. Furthermore, the lipid metabolism transcriptional landscape is emerging as a rich area of research, especially in cancer development and progression [8]. Exploring the mechanistic insights of lipids in liver diseases may open the door to developing effective therapeutics. Because lipids are incorporated into vital functions and inflammatory processes, so they have been considered the first extracellular and second intracellular signals in the cellular recognition process [20]. Although many researchers have investigated how lipid change affects transcriptional processes, the gap remains in our knowledge of how particular lipids modify the metabolic landscape of liver disease. These changes in metabolic status can be tracked by metabolomics analysis. Metabolomics is a comprehensive approach for identifying metabolic signatures towards screening, prediction, and earlier diagnosis with great privilege over conventional diagnostic tools [21].

Moreover, it is essential to understand the disease pathophysiology and better monitor high-risk individuals. The potential of metabolomics as a high-throughput holistic diagnostic platform would avoid the drawbacks of depending on a single metabolite alteration. Therefore, metabolite profiling (metabotyping) is considered a powerful unbiased tool to characterize different pathways involved in disease progression [22].

Based on these facts, we aimed to explore the metabolic signature between NASH and NASH-HCC patients using a TripleTOF 5600+ analytical technology to elucidate the possible reprogrammed metabolic pathways that drive cancer progression in NASH patients.

## 2. Results

### 2.1. Clinical Characteristics of NASH and NASH-HCC Patients

The median age for the entire cohort of NASH and NASH-HCC patients was 62 (range 56.5–67.5) years and 63 (range 58–66) years, of which ~77% and 83.5% were male, and ~23% and 16.5% were female, respectively. Therefore, NASH and NASH-HCC patients exhibited a higher predominance of the male gender than the female gender. The Body mass index (BIM) was higher (overweight) than the normal range (BMI < 25), 30 (28–33.4), and 28.5 (25.7–32) in both NASH and NASH-HCC patients, respectively, as shown in Table 1.

### 2.2. Hematological and Biochemical Analysis 

TAG as a marker for the lipid profile showed an increment in NASH and NASH-HCC 185 mg/dL (range 143–231) and 169 mg/dL (range 155–189), which is higher than the known normal range (less than 150 mg/dL). Prothrombin activity was significantly (*p* = 0.001) decreased in NASH-HCC patients by 52% compared to NASH patients with no significant change in hemoglobin levels. Platelet count (*p* = 0.025) and total leucocyte count (*p* = 0.041) were significantly decreased in NASH-HCC patients by 67% and 69%, respectively, compared to NASH patients. Furthermore, our study showed a significant increase in serum AST levels in NASH-HCC patients by 151% compared to NASH patients, as shown in Table 1; ALT, albumin, and bilirubin showed non-significant change among both groups.

### 2.3. Tumor Markers and Diabetic Profile

Significant (*p* = 0.033) increase in serum AFP levels was detected in NASH-HCC patients by 146% compared to NASH patients, as shown in Table 1. Cancer antigen 19-9 was elevated in NASH-HCC, recording 43.2 ± 4.3 U/mL, which is higher than the accepted upper limit of 37 U/mL, while carcinoembryonic antigen was within the normal range in both groups. Diabetic profiles, including FBG, Insulin, and HOMA-IR, showed a non-significant change

### 2.4. Metabolomic Profiling of NASH and NASH-HCC Patients 

**Figure 1:**
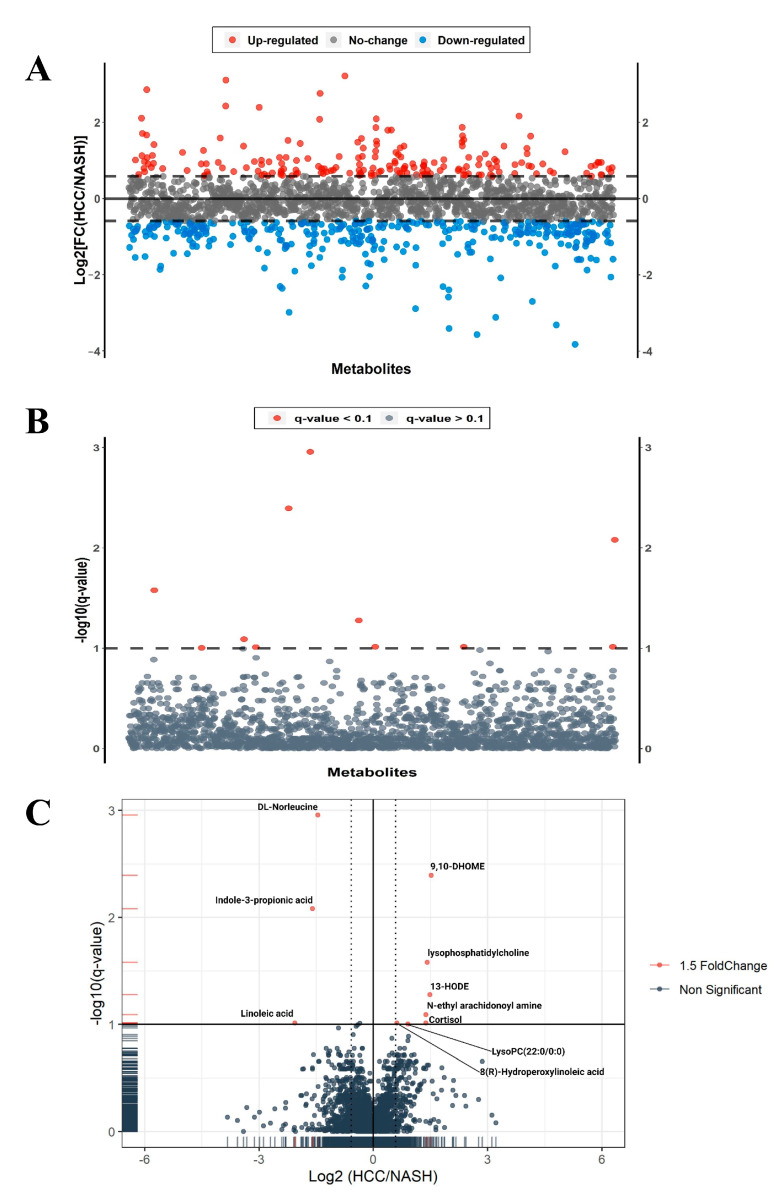
(**A**) Upregulated and down-regulated metabolites with fold change ±1.5 in NASH-HCC patients when compared to NASH patients. (**B**) Wilcoxon (Mann–Whitney) test with FDR 10% (q-value) and *p*-value < 0.05, and 11 metabolites were detected to be significantly (red dots) expressed differently between the two groups. (**C**) Volcano plot with fold change between the groups equal to 1.5 (and −1.5) and alpha value *p* ≤ 0.05.

Investigating the difference between the serum metabolic profile of NASH and NASH-HCC patients identified 1900 metabolites using the HMDB version 4.0 as a search space. As a result of fold change, we reported 186 metabolites upregulated and 364 down-regulated in NASH-HCC patients compared with NASH patients (Figure 1A). Using the Wilcoxon Mann–Whitney test with a *p*-value < 0.05, 11 metabolites were significantly and differently expressed between the two groups (Figure 1B). Volcano scatters plot with fold change between the groups equal to 1.5 (and −1.5), FDR 10% (q-value), and *p* < 0.05 detected ten small molecules when compared between the NASH and the NASH-HCC groups (Figure 1C).

**Figure 2:**
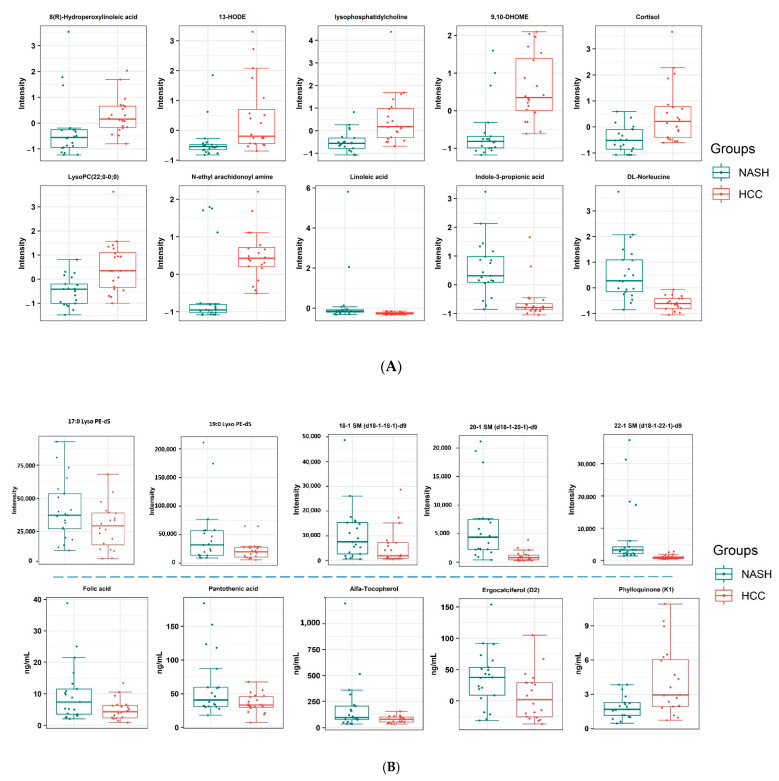
(**A**) Boxplot for the non-targeted molecules in NASH-HCC patients when compared to NASH patients; *p* ≤ 0.05, FDR 10% (q-value), and fold change ±1.5 in non-targeted analysis. (**B**) Boxplot for the targeted molecules in NASH-HCC patients when compared to NASH patients; *p* ≤ 0.05 and fold change ±1.5.

Some of these metabolites were positively associated with NASH-HCC as compared to NASH. They were ordered according to the fold change from the highest to the lowest as 9,10-Dihydroxy-12-octadecenoic acid (9,10-DiHOME), (13S)-Hydroxyoctadecadienoic acid (13-HODE), LysoPC O-15:1, cortisol, N-ethyl arachidonoyl amine, LysoPC 22:6/0:0, and 8(R)-Hydroperoxylinoleic acid (Table 2 and Figure 2A). On the contrary, other significant metabolites were negatively associated with NASH-HCC compared to NASH. These metabolites were ordered from the most significant negative fold change to the lowest as leucine, indole-3-propionic acid, and linoleic acid (Table 2 and Figure 2A).

### 2.5. Targeted Analysis of Lipids 

**Figure 3:**
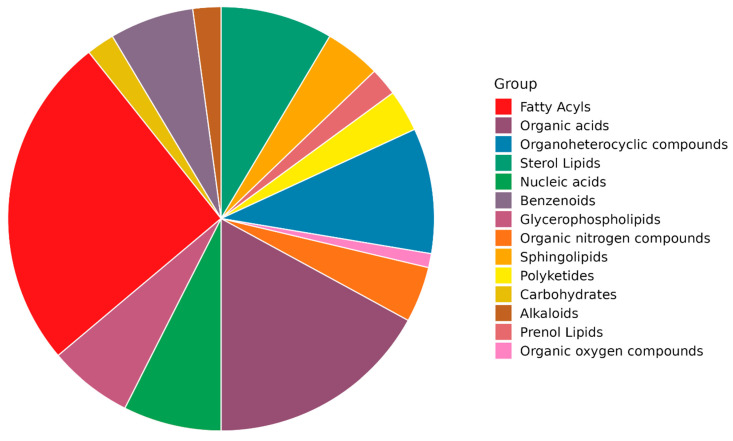
Chemical structure classification of the identified molecules.

We detected high coverage of lipid compounds based on chemical structure classification, as shown in the pie chart (Figure 3). Therefore, we focused on some lipids group analysis, including six groups with twenty deuterated compounds (Supplementary Table S1). Among the analyzed deuterated compounds, five compounds; including 17:0 Lyso PE-d5, 19:0 Lyso PE-d5, 22:1 SM (d18:1/22:1)-d9, 20:1 SM (d18:1/20:1)-d9, 18:1 SM (d18:1/18:1)-d9, showed significant (*p* < 0.05) change with fold change equal to 1.5 (and −1.5) in NASH-HCC patient when compared to NASH group (Table 3 and Figure 2B).

### 2.6. Targeted Analysis of Vitamins and Amino Acids

**Figure 4:**
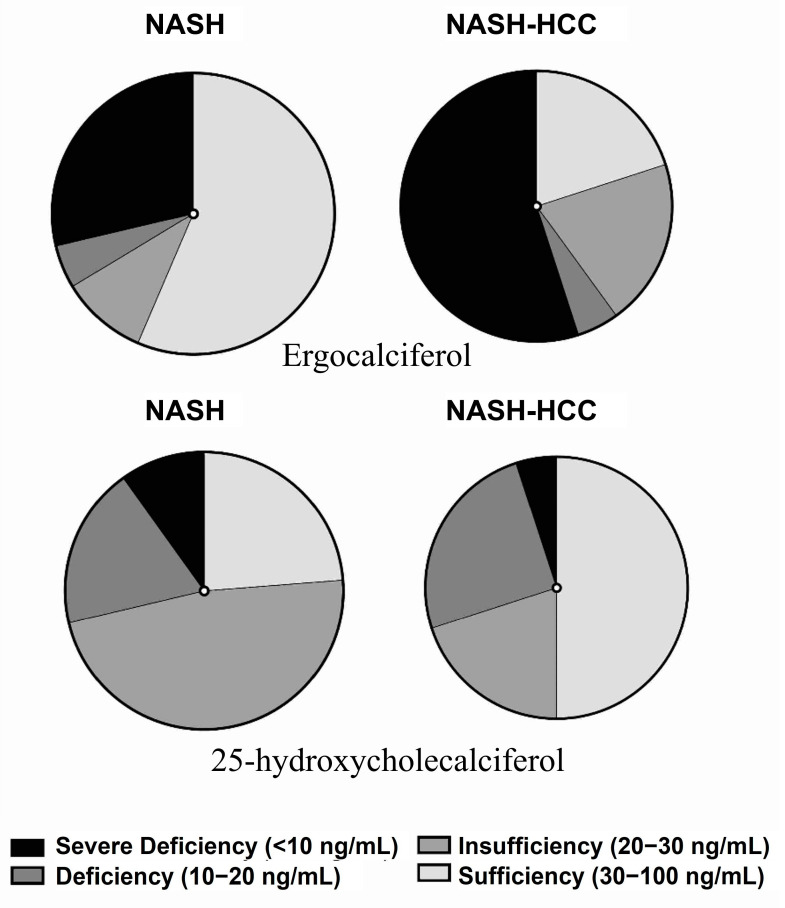
Segregation of ergocalciferol and 25-Hydroxycholecalciferol in the NASH-HCC patients when compared to NASH patients.

Our analysis showed no significant difference in amino acids (L-Phenylalanine and L-Tryptophan). In comparison with the known levels of these two amino acids, we found that their level is lower than the average (Supplementary Table S2 and Figure S1A). Among the panel of measured vitamins, five vitamins were significantly (*p* < 0.05, 1.5 fold change) changed (Table 3); ergocalciferol, α-Tocopherol, phylloquinone, folic acid, and pantothenic acid, as shown in Figure 2B. Ergocalciferol showed a significant decrease in the NASH-HCC group, where 20% only had a sufficient amount compared to NASH 57%. In NASH-HCC patients, 55% of patients showed a severe decrease (<10 ng/mL) when compared to 28.5% in NASH patients (Figure 4). Folic acid and pantothenic acid recorded a significant decrease in NASH-HCC (4.16 and 33.0 ng/mL) patients compared with NASH patients (7.39 and 40.5 ng/mL), respectively. α-Tocopherol showed a significant decrease in NASH-HCC patients (102.4 ng/mL) compared with NASH patients (78.1 ng/mL), and both groups recorded lower levels than the average level in healthy humans. Surprisingly, phylloquinone showed a significant increase in NASH-HCC patients (2.9 ng/mL) compared with NASH patients (1.6 ng/mL), as shown in Figure 2B. These findings were compared with the previous studies, which are shown in Supplementary Table S2 and Figure S1B. The results clarified that the level of the vitamins was almost average except for pantothenic acid, which showed a lower level than the average concentrations (Supplementary Table S2).

### 2.7. Targeted Analysis of Adenosine, ATP, and 5′-AMP

**Figure 5:**
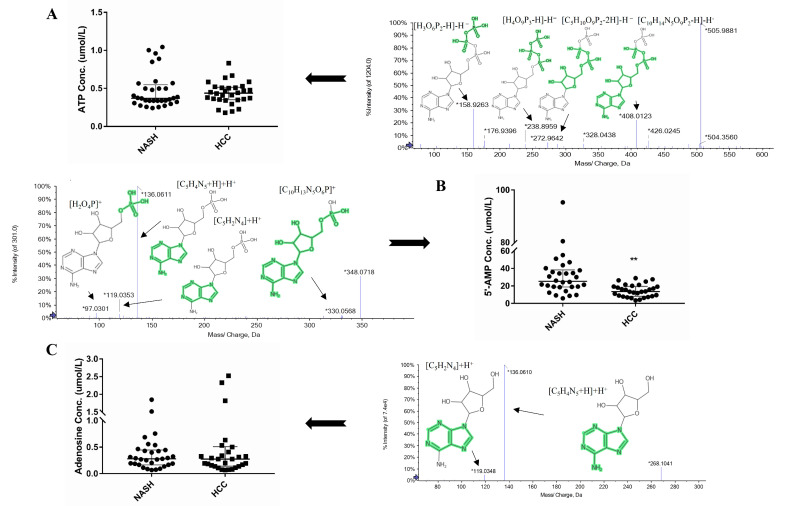
Quantification of (**A**) ATP, (**B**) 5′-AMP, and (**C**) adenosine in NASH and NASH-HCC patients with fragmentation pattern for each compound. ** *p* < 0.01 in the boxplot.

As per remodeling of mitochondrial energetics, which derives the progression of NASH into HCC, ATP, 5′-AMP, and adenosine levels were determined, and results showed that 5′-AMP was significantly (*p* < 0.05) downregulated in NASH-HCC (13.6 µmol/L) as compared to NASH (25.3 µmol/L) patients (Figure 5). Meanwhile, adenosine and ATP concentration showed a non-significant change among tested patients (Figure 5).

### 2.8. Metabolomics and Clinical Data Clustering between NASH and NASH-HCC Patients 

**Figure 6:**
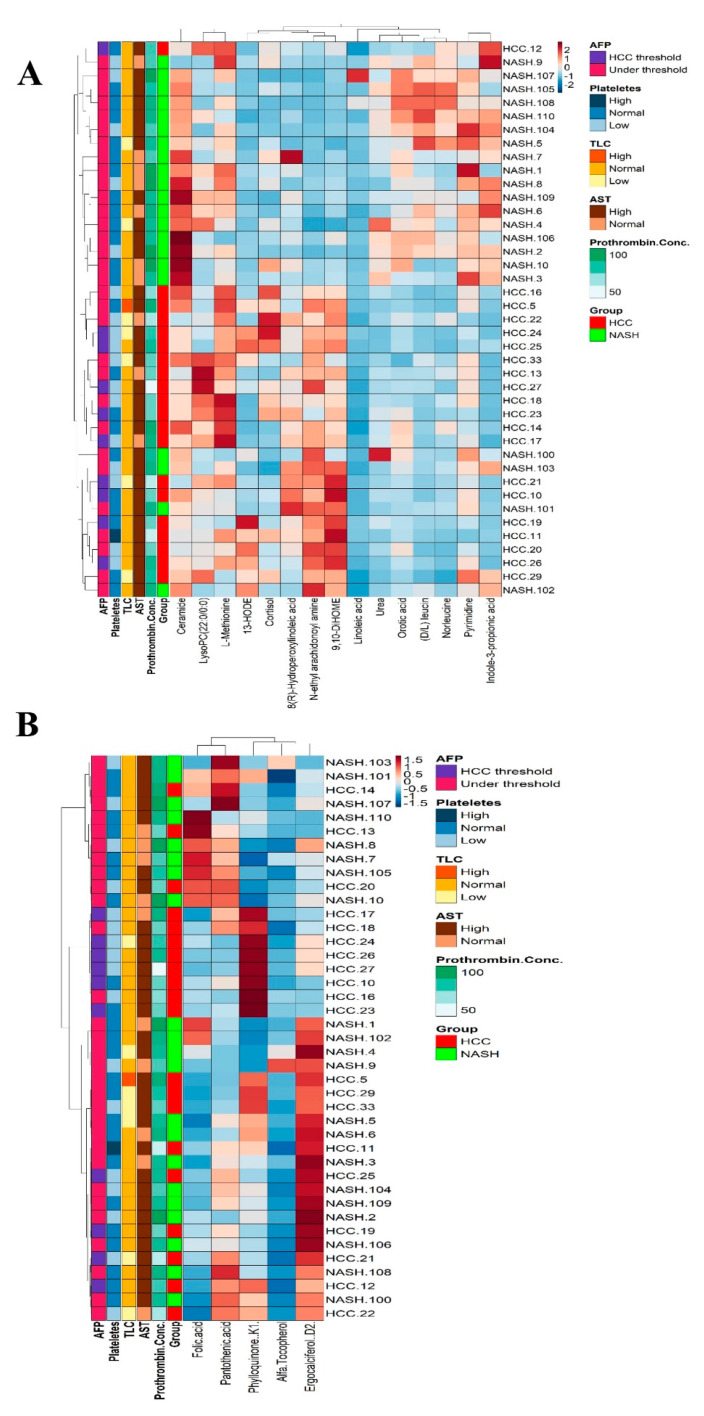
(**A**) Heatmap analysis showing the significant metabolites intensity for all samples with clustering. (**B**) Heatmap for upregulated and down-regulated vitamins in the NASH-HCC patients when compared to NASH patients.

The correlation matrix between the top 15 non-target metabolites showed significant segregation for almost all samples, as illustrated by the heatmap chart (Figure 6A). One NASH-HCC sample was not assembled in the NASH-HCC direction. On the other hand, the biochemical and hematological parameters in the heatmap clarified that platelet count, TLC, PT, and AST failed to completely segregate NASH and NASH-HCC patients (Figure 6A). Furthermore, the heat map highlighted that AFP could not completely segregate NASH from NASH-HCC patients compared to identified metabolites (Figure 6A). AFP recorded the HCC threshold in only 50% of the NASH-HCC patients. TLC and platelet count were within the normal range in both groups. AST and prothrombin activiy were directed to the higher level in both groups. Water- and fat-soluble vitamins could segregate 60% of the NASH patients from the NASH-HCC patients (Figure 6B).

### 2.9. Pathway Enrichment Analysis 

**Figure 7:**
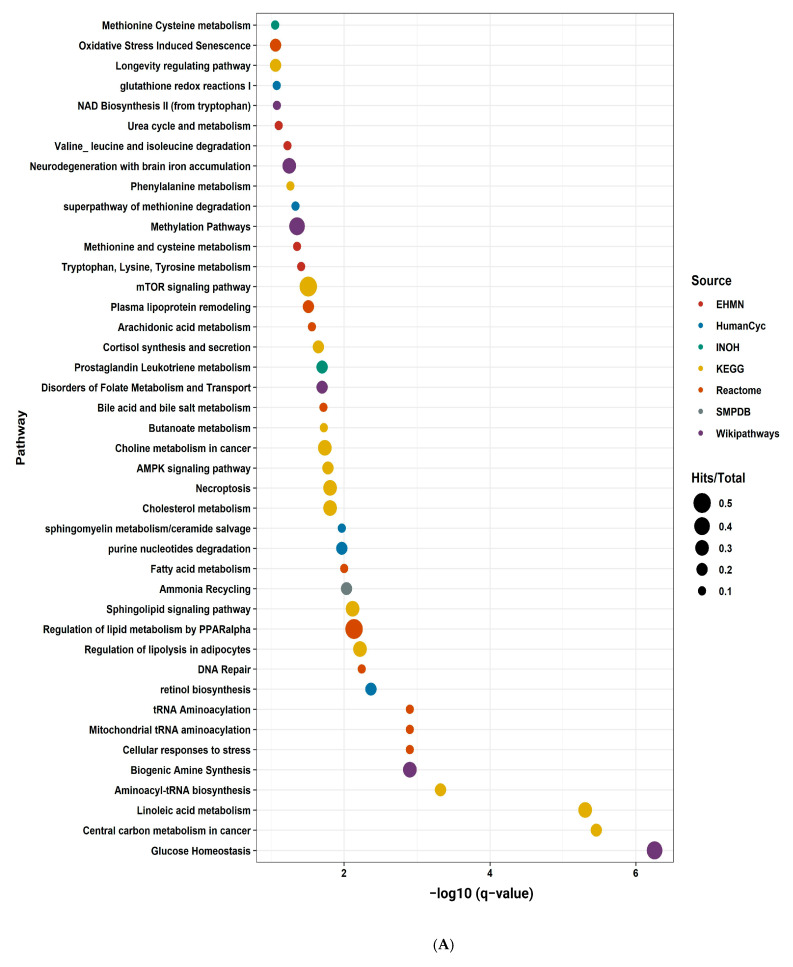

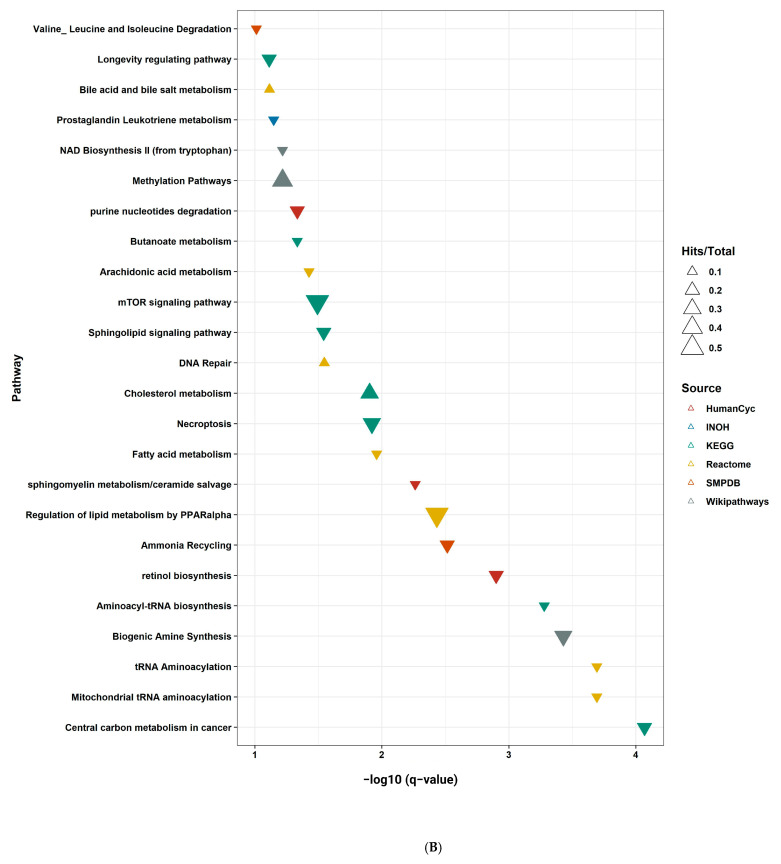
(**A**) Pathway enrichment analysis with FDR 10% (q-value) and *p*-value < 0.05 using different sources as background database search. Each database is illustrated with a color code in the figure legend. The size of the circle reflects the number of metabolites found in this pathway over the total metabolites. The x-axis is the −log10 of the q-value. This analysis includes both up- and down-regulated metabolites with a fold change cut-off equal to 1.5. (**B**) Pathway enrichment analysis with FDR 10% (q-value) and *p*-value < 0.05 using different sources as background database search. Each database is illustrated with a color code in the figure legend. The size of the arrows’ head reflects the number of metabolites found in this pathway over the total metabolites. The x-axis is the −log10 of the q-value. This analysis includes up- or down-regulated metabolites with a fold change cut-off equal to 1.5, as described in the Materials and Methods. If the head of the arrow point is upward, that pathway is only enriched using the upregulated metabolites. If the head of the arrow is downwards, that pathway is only enriched using the down-regulated metabolites.

A pathway enrichment analysis was conducted using Integrated Molecular Pathway Level Analysis (IMPaLA) for NASH and NASH-HCC. Pathway enrichment analysis was performed with FDR 10% (q-value), fold change cut-off equal to 1.5, and *p*-value < 0.05 using different sources as a background database search. Significant pathways were de tected, as explored in Figure 7A. These pathways were related to the transport of amino acid and fatty acid metabolism, bile salts and organic acids, oxidative stress, glucose homeostasis, ammonia recycling, and the urea cycle. Among the assigned down-regulated pathways in NASH-HCC compared to NASH patients were necroptosis, amino acid metabolism, regulation of lipid metabolism by PPAR-α, biogenic amine synthesis, fatty acid metabolism, and mTOR signaling pathway (Figure 7B). On the other hand, cholesterol metabolism, DNA repair, methylation pathway, and bile acid and salts metabolism were significantly upregulated in NASH-HCC compared to the NASH group (Figure 7B and Supplementary Table S3).

### 2.10. An Integrative Metabolite–Protein Interactions Network

OmicsNet was used to generate a direct Metabolite–Protein network association using significant fold change (1.5 FC) features. The network consists of nodes representing metabolite and protein biological entities (Figure S2 and Supplementary Table S4). Connecting these nodes infer direct molecular interactions. Many well-known proteins that play crucial roles in cancer, including purine nucleoside phosphorylase (PNP), Phosphatidylethanolamine N-methyltransferase (PEMT), CDK5 Regulatory Subunit-associated Protein 1-Like 1 (CDKAL1), Betaine-homocysteine S-methyltransferase (BHMT2), glutamic-oxaloacetic transaminase family (GOT1, GOT2), Trans-Activator of Transcription (TAT), BAAT (Bile Acid-CoA: Amino Acid N-Acyltransferase), Nicotinamide N-Methyltransferase (NNMT), Phosphatidylethanolamine N-methyltransferase (PEMT), N-Acylsphingosine Amidohydrolase (ASAH1-2, Alkaline Ceramidase 1-2 (ACER1-2), CYP4F11, Cytosolic phospholipase A2 beta (PLA2G4β), L-amino-acid oxidase precursor (IL4I1), and the adenosyl homocysteinase family (AHCYL, AHCYL 1, AHCYL2) were represented in this network. Some metabolite-interacting proteins participated in other aspects such as immune checkpoints, energy metabolism, and ROS balance, highlighting that metabolites can participate in various cellular pathways via interactions with various proteins that may favor carcinogenesis and tumor aggressiveness.

## 3. Discussion

Thought to appear benign, NASH involvement in HCC development exceeds hepatitis viruses’ contribution, especially with the contemporary lifestyle and NASH patients not periodically screened for HCC. Thus, understanding NASH metabolic reprogramming, the core of HCC progression, becomes increasingly urgent [8]. Our study aimed to compare the metabolic signature between NASH and NASH-HCC patients, which could determine robust markers that might drive HCC progression from NASH. Although AFP was the only reported serum marker, recent reports highlighted that increased AFP levels are only elevated in about one-third of the NASH-HCC patients [23]. This finding aligns with our AFP results, which failed to segregate NASH from NASH-HCC patients completely. Although a significant increase was detected in serum AFP levels in NASH-HCC patients compared to NASH patients, there are wide ranges between NASH-HCC individuals (5.1–882 ng/mL). Therefore, AFP presented restricted potentiality for differentiating NASH-HCC from NASH patients [8]. Furthermore, there was a significant increase in serum AST levels in NASH-HCC patients by 151% compared to NASH patients, despite being suggestive of liver damage but not specific to hepatic carcinogenesis. In addition, our results showed that other diabetic profiles such as FBG, Insulin, and HOMA-IR and biochemical and hematological parameters such as platelet count, TLC, PT, and AST also failed to segregate NASH and NASH-HCC patients [8]. 

Thus, we aimed for a non-invasive metabolic signature that compares NASH and NASH-HCC patients to explore the reprogrammed metabolic pathways that drive cancer progression in NASH patients. In this context, our results showed a pattern of 20 metabolites from targeted and non-targeted analyses that could segregate NASH-HCC from NASH patients. Transnationally, these disturbed metabolites reflect the activation or downregulation of some significant pathways in NASH-HCC compared to NASH patients. These pathways were related to the transport of amino acid metabolism, fatty acid metabolism, bile salts and organic acids metabolism, oxidative stress-induced senescence, glucose homeostasis, ammonia recycling, urea cycle, and iron accumulation. Among the assigned pathways significantly downregulated in NASH-HCC compared with NASH patients were necroptosis, amino acid metabolism, and regulation of lipid metabolism by PPAR-α, biogenic amine synthesis, fatty acid metabolism, and mTOR signaling pathway.

On the other hand, cholesterol metabolism, DNA repair, methylation pathway, and bile acid and salts metabolism were significantly upregulated in NASH-HCC compared to the NASH group. A significant increase in cortisone, turn cortisol synthesis, and excretion pathway in NASH-HCC patients was reported. Previously, it was anticipated that NAFLD metabolically affects steroid metabolism [24]. The deranged cortisol/cortisone shuttle increases glucocorticoids with further metabolic consequences of insulin resistance and hypertriglyceridemia [24]. Elevated serum corticosteroids are associated with an increased risk for HCC development in autoimmune chronic active hepatitis [25].

Moreover, microbial metabolites have emerged as critical components that mediate metabolic effects on the liver through the “gut–liver axis” [26]. Thereby, gut bacterial dysbiosis is associated with worsening liver disease through enterohepatic circulation [27]. Indole-3-propionic acid, a deamination metabolite of tryptophan produced by gut bacteria, can increase the diversity of intestinal microbes and maintain intestinal homeostasis through the induction of tight junction protein expressions, such as ZO-1 and Occludin [28]. Current results revealed a significant downregulation of Indole-3-propionic acid in NASH-HCC patients. Previously, Indole-3-propionic acid was reported to differentiate NASH patients from alcohol-related liver damage and was considered a potent anti-NASH and -HCC microbial metabolite in rats fed a high-fat diet [29]. Indole-3-propionic acid prevents lipid peroxidation and acts as a free radical scavenger, the primary causative factor of liver inflammation and damage [30].

Moreover, Indole-3-propionic acid has the ability to inhibit the expression of fibrogenic genes such as transforming growth factor-β (TGF-β) [30], α-smooth muscle actin (α-SMA), and collagen synthesis genes that activate ROS [28]. Therefore, it directly impacts the metabolic and antioxidant function of the mitochondria [31]. Among the unregulated pathway in NASH-HCC are cholesterol and bile acid metabolism. Cholesterol is considered the primary lipotoxic molecule that promotes the progression of NASH and liver cancer [32,33]. Depletion of Indole-3-propionic acid was noted in hypercholesterolemia-fed HCC mice [33]. Furthermore, Indole-3-propionic acid could inhibit the accumulation of triglycerides in the cholesterol-induced human hepatocyte cell line and inhibit the proliferation of NASH-HCC cell lines [33]. Moreover, another study clarified that indole-3-propionic acid reduced fatty acid transcription genes in HepG2 cells [34]. Therefore, the damage to the tryptophan metabolism and the reduction in serum indole-3-propionic acid content promotes the development of NASH-HCC.

Advanced liver diseases are commonly associated with amino acid imbalance, affecting patients’ prognosis. Leucine and isoleucine are essential branched-chain amino acids. In the current study, we reported a decreased level of leucine in NASH-HCC compared with NASH patients, and pathway analysis showed a significant upregulation of leucine and isoleucine degradation pathways. Leucine, a metabolic regulator, was associated with HCC development and progression [35]. Thus, it might lie at the intercept of NASH-dysregulated metabolism and cancer progression, as previous reports associated its level with liver inflammation and carcinogenesis [35]. Previously, branched-chain amino acids suppressed the growth and angiogenesis of HCC [36] by degrading vascular endothelial growth factor mRNA [37] and inhibiting insulin resistance [36]. A previous study reports the inhibitory effect of these amino acids in obesity-related HCC by suppressing cellular proliferation by interrupting adipokines’ stimulatory effect [38]. They induce apoptosis of liver cancer cell lines by interfering with mTOR-dependent mechanisms [39]. Different research clarified that branched-chain amino acids supplementation reduces the incidence [40,41] and recurrence of HCC by 25–30% and 55.7% [42], respectively. In target analysis, even though our data showed no significant difference in amino acids (L-Phenylalanine and L-Tryptophan) among both diseased groups, we compared our results with the known levels of these two amino acids, and we reported a decrease in both of them from the average level. Both phenylalanine and tryptophan are involved in various biological functions, including protein synthesis, metabolism, and degradation [43]. Tryptophan is a precursor of serotonin and melatonin, while phenylalanine is a precursor of tyrosine, dopamine, and epinephrine [43]. Previously, Vanessa et al. reported an elevation in the level of tryptophan and phenylalanine in the NASH group compared to those with normal liver. This group assumed this elevation was due to impaired hepatic metabolism [44].

On the other hand, some researchers clarified that the consumption and metabolism of amino acids are the most demanding and leading substrates for biological processes needed for cancer cell growth, especially tryptophan, tyrosine, phenylalanine, methionine, isoleucine, leucine, and glutamine [45]. This massive amino acid consumption, in turn, lowers their levels. Moreover, they reported that the most consumed amino acid is leucine, which is heavily involved in the human proteome, while tryptophan is the least [45]. In line with this, we report marked downregulation in leucine but not in tryptophan. Although therapeutic use of amino acids in patients with liver diseases is promising, scientists are divided between supporters and opponents of their use due to the discrepancy in the previous study results, so therapeutic use of amino acids must be performed carefully, with further studies still needed to select the proper type and dose of amino acid and stage of liver disease. 

Evidence that links linoleic acid, an essential polyunsaturated fatty acid, with carcinogenesis is still controversial. Our study showed a significant depletion of linoleic acid in NASH-HCC compared to NASH patients. Previous evidence points to the association of linoleic acid depletion with HCC progression from different sources, either NAFLD-HCC base [8,46] or viral-associated HCC [47]. Moreover, linoleic acid accumulation causes suppression of colorectal cancer [37]. The theory behind its suppressive effect is due to its vital role in inflammatory response through inducing reactive oxygen species, apoptosis, and mitochondrial dysfunction [48]. Linoleic acid is a precursor to arachidonic acid, a substrate in prostaglandin synthesis; therefore, a decrease in linoleic acid may result in inflammatory signal disturbance [36]. Conversely, in a mouse model, accumulation of linoleic acid causes CD4+ T cell loss and reactive oxygen species increment, leading to NAFLD-mediated liver carcinogenesis [49]. Interestingly, Lu et al. reported that linoleic acid at high concentrations >300 μM suppresses tumor growth, while at low concentrations (100–200 μM) it enhances proliferation [48], which explains the contradiction in the different studies. Collectively, augmentation of linoleic acid might be a potential HCC therapeutic target.

The lipid metabolism transcriptional landscape is emerging as a rich area of research, especially in cancer development and progression. Lipids are incorporated in vital functions, including energy storage, inflammatory processes, and cellular recognition, and they are considered first as extracellular and second as intracellular signals in the recognition process [20]. Although many researchers have investigated how lipid changes affect transcriptional processes, the gap remains in our knowledge of how particular lipids modify the metabolic landscape of liver disease. In the current study, some lipids were positively associated with NASH-HCC compared to NASH in both targeted and non-targeted analyses. The imbalance between fatty acid uptake, fatty acid oxidation, and secretion leads to lipid accumulation and disease progression [50]. Sphingomyelin (SM) is the most abundant sphingolipid, essential for creating sterol-enriched membranes and cell signaling [51]. In alignment with our result, Zheng et al. reported that the risk of non-alcoholic fatty liver disease was increased with higher plasma phospholipids [52]. Non-alcoholic fatty liver disease with hyperglycemia reported increasing circulating phosphatidylethanolamine [53]. Previously, phospholipids were depleted in patients with NASH [54]. This may be due to the enzymes that convert lysophospholipids into phospholipids in low abundance in NASH patients. This point may clarify the accumulation of lysophospholipids in the current study with the progression of NASH toward HCC. Phospholipids can act as signal transductors with oncogenic and tumor-suppressive roles but not lysophospholipids, which may increase hepatic cancer cell migration and invasion [55]. Lysophosphocholin has controversial results. A higher level was reported in NASH patients [56].

Conversely, in vitro research reported increased or unchanged levels in NASH patients’ livers and found that lysophosphocholin activates apoptosis by activating the tumor necrosis factor-α pathway [57,58]. A previous study reported the depletion of sphingomyelins due to the activation of its conversion to ceramides, a process catalyzed by sphingomyelin phosphodiesterases, but the opposite direction was repressed [8]. They reported that HCC from different cancer origins has different lipidomics patterns; there was a decrease in sphingomyelin in HCC from the viral origin compared to from NAFLD origin [8]. Supportively, we reported sphingomyelin downregulation in NASH-HCC patients. Cooperatively, our findings showed significant downregulation in sphingomyelin metabolism/ceramides salvage. Another interesting finding was that eicosanoids, especially 9,10-DiHOME and 13-HODE, were elevated in the sera and liver of HCC patients [59], which agrees with our results. DiHOMEs play a dual role in inflammation, activating neutrophil chemotaxis at low concentrations while inhibiting neutrophil respiratory burst at higher concentrations [59]. Another aspect, disruption of lipid metabolism, is one of the major hallmarks of NASH and its progression to HCC [60]. Accumulating hepatic fatty acids in NASH results in decreased mitochondrial β-oxidation with increased peroxisomal and microsomal ω-oxidation, activating the lipotoxicity state and disease progression [60]. Since there are controversial results, more studies focusing on lipidomics analysis are still needed to resolve this apparent paradox and explore the underlying mechanism in different disease stages.

Finally, our metabolite–protein network association showed proteins playing roles in HCC progression as PEMT, PLA2G4β, IL4I1, ACER, BAAT, TAT, GOT, CDKAL1, PNP, NNMT, NOS1, and AHCYL [61,62]. Being involved in the genetic, epigenetic, genesis, invasion, and metastasis of HCC, our study presented these candidate proteins associated with NASH-HCC, which warrants future functional studies to study their precise role. This integrated bioinformatics analysis with a comprehensive analysis of metabolic alterations, including protein interactions, will present multiple candidate biomarkers and novel therapeutic targets for the prognosis of the disease and the identification of targets at the molecular and pathway levels. Higher PEMT activity may be associated with breast and liver tumors’ aggressiveness due to its role in lipid metabolism necessary for highly proliferating cancer cells [63].

## 4. Materials and Methods

### 4.1. Chemical and Reagents

Twenty isotope-labeled standards, including Lyso PI-d5, Lyso PS-d5, Lyso PG-d5, Lyso PC-d5, Lyso PE-d5, sphingomyelins-d9 families, were purchased from Avanti Polar Lipids Inc., Alabaster, AL, USA (Supplementary Table S1). Authentic standards of seven water-soluble vitamins, six fat-soluble vitamins, and two amino acids were purchased from Sigma-Aldrich Co., St. Louis, MO, USA (Supplementary Table S1). Adenosine, adenosine triphosphate (ATP), and 5′-Adenosine monophosphate (5′-AMP) standards were obtained from Sigma-Aldrich (Sigma-Aldrich Co., St. Louis, MO, USA). LC-MS grade solvents, including acetonitrile, isopropanol, methanol, and dichloromethane, were purchased from Thermo-Fisher (Thermo Fisher Scientific, Waltham, MA, USA). Other solvents, such as formic acid, ammonium hydroxide, ammonium formate, and chloroform, were purchased from Sigma-Aldrich (Sigma-Aldrich Co., St. Louis, MO, USA). Millipore (Burlington, MA, USA) provided Milli-Q water.

### 4.2. Study Population and Sample Collection

A total of 61 patients were included in this study: 31 NASH patients and 30 NASH-HCC patients from a NASH background were recruited from the Liver Diseases and Research Center in Cairo and the National Liver Institute in Menofia, Egypt, respectively. Informed consent was obtained from each patient prior to sample collection. The Research Ethics Committee of the Faculty of Pharmacy, Ain Shams University approved this study under approval no. ENREC-ASU-66.

Patients in this study were diagnosed based on biopsy sampling and radiological evidence following the guidelines of the European Association for the Study of the Liver (EASL) [64]. The NASH group was diagnosed through radiological evidence of hepatic steatosis, where the severity of steatosis was scored according to the validated Kleiner criteria [65]. The unweighted sum of steatosis, lobular inflammation, and hepatocellular ballooning scores, NAFLD activity score (NAS), was used to address the full spectrum of lesions of NAFLD. In the current study, NASH patients have a score of 5, which correlates with a diagnosis of NASH [65,66]. The NASH group was non-cirrhotic. For NASH-HCC, they were cirrhotic according to METAVIR score [67].

The exclusion criteria included viral hepatitis, autoimmune diseases, and hepatic toxicity due to drugs and/or alcohol consumption. All samples were collected and stored according to REMARK for biomarker analysis [68]. Patient characteristics are summarized in Table 1. Fasting blood samples were collected and separated for hematological, biochemical, targeted, and non-targeted metabolomic analyses. Whole blood and serum samples were collected from each patient. The whole blood was used for hematological analysis. Blood samples with sodium citrate were used for prothrombin activity. Serum samples were separated by samples centrifugation at 4000 rpm for 20 min at 4 °C. Aliquots were prepared and stored at −80 °C for subsequent biochemical and metabolomics analysis.

### 4.3. Blood Analysis

#### 4.3.1. Hematological and Biochemical Test

Hemoglobin content, platelet count, and total leukocyte count were measured in 31 NASH and 30 NASH-HCC patients. The biochemical analysis included total bilirubin, serum albumin, serum triacylglycerol (TAG), and serum transaminases (ALT, AST) were determined by enzymatic methods using an autoanalyzer (Beckman Synchron cx systems, Beckman Coulter Inc., Brea, CA, USA) [69]. Blood samples with sodium citrate were used for prothrombin time by electromechanical detection [70,71].

#### 4.3.2. Diabetic Profile

Serum insulin in 31 NASH patients and 30 NASH-HCC patients was quantified using the ELISA technique using the commercially available kit (Nova Tec Immundiagnostica GmbH, Dietzenbach, Germany). Fasting blood glucose (FBG) was determined using the automated biochemistry analyzer’s Dimension RxL analyzer (Dade Behring, Newark, DE, USA). The following equation calculated Homeostatic Model Assessment for Insulin Resistance (HOMA-IR): HOMA-IR = fasting insulin (µU/mL) × FBG (mmol/L)/22.5 [72].

#### 4.3.3. Tumor Markers

Serum alpha-fetoprotein (AFP) in 31 NASH patients and 30 NASH-HCC patients was quantified by EIA using the available kit (Human CanAg AFP, Fujirebio, Diagnostic, Inc. Goteborg, Sweden. Carcinoembryonic antigen (CEA) and cancer antigen 19-9 (CA 19-9) were quantified using ELISA assay kits (Thermo Scientific, Santa Clara, CA, USA).

### 4.4. Samples and Standards Preparation

#### 4.4.1. Sample Preparation for Metabolomics Analysis

Forty-one serum samples (21 NASH and 20 NASH-HCC) randomly selected out of a total of 61 collected serum samples were thawed on ice and aliquoted into 50 µL. Briefly, 300 μL of a precooled extraction solvent was added to the samples. The extraction solvent used for metabolites extraction consists of chloroform: methanol: water at a ratio of 1:3:1, respectively [73]. The latter efficiently extracts hydrophilic and mid-hydrophobic metabolites [74,75]. The mixture was vortexed and whirled for at least 10 min. Samples were ultra-sonicated for 15 min at 20 °C. Samples were centrifuged at 12,000 rpm at 4 °C for 10 min. The supernatant (250 μL) was transferred to a clean Eppendorf tube. Samples were further dried using a speed vac at 30 °C and reconstituted in a solvent consisting of water: methanol: acetonitrile at a ratio of 2:1:1, respectively. The extracted samples were subjected to LC-MS/MS analysis. A pooled quality control (QC) was validated by mixing 5 µL of each sample. The QC samples were injected at the beginning, end, and every ten injections in the sequence. The QC sample was run to check technical reproducibility and RSD%. Mass calibration was automatically performed every 2 h by an automated calibration delivery system using either positive or negative APCI calibration solution (AB SCIEX). The blank sample was used to check the solvents’ quality and monitor possible carryover.

#### 4.4.2. Standards Preparation

According to the Bioanalytical Method Validation, a previously validated in-house library integrated into MasterView 1.1 software (AB SCIEX) was used for small molecules targeted detection. Standards for L-Amino acids (L-Phenylalanine and L-Tryptophan; Cat. No. LAA21-1KT), nucleotides (adenosine, ATP, and 5′-AMP), water-soluble vitamins (folic acid (47866), pyridoxine hydrochloride (47862), D-Pantothenic acid (47867), nicotinic acid (47864), nicotinamide (47865-U), riboflavin (47861), thiamine hydrochloride (47858)), and fat-soluble vitamins (retinol (R7632-25MG), 25-hydroxycholecalciferol (47763), ergocalciferol (47768), α-Tocopherol (47783), γ-Tocopherol (47785), and phylloquinone (47773)) were used (Supplementary Table S1). The working solution concentration ranges of the calibration curve were 0.5–2048 ng mL^−1^ for all standards except for riboflavin, thiamine, L-Tryptophan, and L-Phenylalanine, where the calibration solution was up to 7.8 µg mL^−1^. The working solution was prepared by taking the appropriate concentration of each stock solution and reconstituting it in a solvent consisting of water:methanol:acetonitrile 2:1:1, respectively. Adenosine, ATP, and 5′-AMP working solutions were prepared in methanol:water (1:1) [76]. Their calibration range was 8–4096 ng mL^−1^. The calibration curves were determined using the peak area of each analyte versus the nominal concentrations by a least-squares regression model. The regression coefficient ranged from 0.96 to 0.99. The lowest quantification concentration of the calibration curve was accepted as LLOQ. The carryover effects were evaluated by analyzing the background matrix before and after the injection of the upper limit of quantification samples. The residues were less than 15% of LLOQ. NASH and HCC-NASH sample concentrations were detected by comparing peak area versus each standard, as explained by Bell, et al. [77]. A predefined library for targeted lipidomics detection of 20 deuterated standards, including Lyso PI-d5, Lyso PS-d5, Lyso PG-d5, Lyso PC-d5, Lyso PE-d5, SM-d9 families, were analyzed depending on their fragmentation pattern. Deuterated standards concentrations are available in Supplementary Table S1.

### 4.5. LC-MS/MS Analysis Using the SWATH Acquisition Method

#### 4.5.1. LC Method

Chromatographic separation was carried out using a SCIEX EXION LCTM AC UHPLC system using a Phenomenex In-Line filter disk 0.5 µm × 3.0 mm equipped with Acquity XSelect HSS T3 analytical column 2.1 × 150 mm, 2.5 µm ID maintained at 40 °C (Waters Co., Milford, MA, USA). The XSelect HSS T3 is compatible with 100% aqueous mobile phase and can separate polar and non-polar compounds.

#### 4.5.2. Mass Spectrometry Method

Mass spectrometric analysis was performed using a Triple TOFTM 5600+ system (AB SCIEX, Concord, ON, Canada) equipped with a Duo-Spray source operated using the positive-ion (ESI+) and negative-ion (ESI−) modes to detect many ions. The ion spray voltage capillary was 4500 eV with declustering potential voltages of 80 V in positive and −80 V in negative modes. The source temperature was set at 600 °C, the curtain gas was 25 psi, and gas 1 and 2 were 40 psi. The collision energies were 35 V and −35 V for positive and negative modes, respectively, with CE spreading 20 V. Sequential windowed acquisition of all theoretical MS (SWATH) methods was utilized to capture all metabolites within the samples [78,79]. The SWATH Acquisition method consisted of a single TOF scan from 50 to 1100 Da accumulated in 30 ms and a fragment ion TOF scan from 50 to 1100 Da using fixed 50 Da transition windows [78]. Batches for MS and MS/MS data collection were created using Analyst TF (v 1.7.1).

#### 4.5.3. Sample Processing

Extracted samples were kept at 8 °C during analysis, and 10 µL of each sample was injected for 28 min of gradient elution with a flow rate of 0.3 mL/min. Samples were injected twice in random order with QC runs in between. The mobile phase solutions consisted of solution (A); 5 mM ammonium formate in 1% methanol (pH 3.0) for positive mode, solution (B); acetonitrile, and solution (C); 5 mM ammonium formate in 1% methanol (pH 8.0) for negative mode elution. Gradient elution was sustained at 0% B for 1.0 min, 0% to 90% B in 20 min, 90% for 4.0 min, 90% B to 0% B in 1.0 min, and finally re-equilibrating with 0% B for 3.0 min.

### 4.6. Metabolomics Data Analysis

#### 4.6.1. Non-Targeted Analysis

The MS-DIAL platform was used for small molecule identification [80]. High-resolution Human Metabolome Database (HMDB) version 4.0 was used as a search space. For HMDB, relative intensities per component fragments SPLASH (SPectraL hASH; database-independent identifier) were filtered and stratified depending on their relative intensity. The latter with relative intensity greater than 75% were considered the main fragment (base peak), while fragments with relative intensities lower than 5% were excluded. Fragments >5% and <75% were considered as secondary fragments. Manual validation was performed to confirm ideal alignment using PeakView 2.2 with MasterView 1.1package (AB SCIEX) to give more credence to the identified molecules. The mass shift was 10 ppm, and the sample’s signal was at least five times higher than the blank. The identified small molecules were used for subsequent statistical analysis and biological evaluation.

#### 4.6.2. Targeted Analysis

Targeted metabolomics analysis of 20 deuterated standards, including Lyso PI-d5, Lyso PS-d5, Lyso PG-d5, Lyso PC-d5, Lyso PE-d5, and SM-d9 families, were analyzed depending on their fragmentation pattern. Moreover, a set consisting of thirteen vitamins (fat- and water-soluble vitamins), two amino acids, and three nucleotides was quantified. Metabolites annotation was performed by comparing the mass spectrum to the available library using MasterView 1.1 software and MultiQuant 3.0.2 Software (AB SCIEX). Standards concentrations in NASH and NASH-HCC samples have been calculated in each sample as per area under peak estimated from the calibration curve, as explained by Bell, John, Hughes and Pham [77].

### 4.7. Pre-Processing and Statistical Analysis

#### 4.7.1. Data Pre-Processing

After exporting the alignment and identification file from MS-DIAL, each group (NASH and NASH-HCC) was treated separately. Metabolite features with more than 50% missing values were removed per group. Then, an imputation method was employed on the rest of the features [81,82]. Median random imputation, a range of ±10% around the median, is generated. Each time a missing value is imputed, a random value from that range is assigned. If the median of the feature is equal to zero, the missing value is automatically set to zero [81,82]. This approach keeps the feature median in a group and prevents tied observations in the data. Features along samples, in both groups, assigned to abundance < 1000 in more than 50% of the samples were removed from final analyses.

#### 4.7.2. Data Normalization and Transformation

Before statistical analysis, data normalization using Probabilistic Quotient Normalization (PQN) starts with the calculation of a reference spectrum based on the median spectrum [83], and auto-scaling (mean-centered and divided by the standard deviation of each variable) was performed.

#### 4.7.3. Statistical Analysis

To detect the statistically significant features of metabolites between the two groups, the Wilcoxon Mann–Whitney test was used with a false discovery rate (FDR) of 10% [84,85,86] and *p*-value < 0.05, with a fold change cut-off equal to 1.5 (NASH-HCC/NASH). The data were identified as nonparametric using the Shapiro–Wilk test. Further, multivariate statistical analyses were performed, including Principal Component Analysis (PCA). Cluster analysis using only significant features was applied to the data using Euclidean distance and Complete-linkage clustering, represented as a heatmap along with the clinical data. All calculations were performed using MetaboAnalyst R [87] in the R environment, and the ggplot2 package was used for graphical visualization in the study.

### 4.8. Pathway Enrichment Analysis and Metabolite–Protein Knowledge-Based Integration

Pathway enrichment analysis with FDR 10% and *p*-value < 0.05 was performed using IMPaLA on three different metabolite sets [88]. The first set includes up- and down-regulated metabolites with a fold change cut-off of 1.5. Only upregulated and downregulated metabolites were used in the second and third sets, respectively. Only the un-overlapped pathways from the last two sets were considered putative up or down-regulated. A metabolite–protein knowledge-based integration network was performed using a web-based tool, OmicsNet 2.0. Small molecules with a 1.5 fold change threshold were used for integration, and only nodes with a *p* value cut-off <0.05 were kept. The identified molecules’ chemical structures were classified using MetaboAnalyst R [87].

## 5. Conclusions

Using the metabolomics and lipidomics approach, a paradigm metabolic bio-signature was identified that could differentiate NASH from NASH-HCC. This study provided for the first time a detailed map of the most predominant perpetuated metabolic pathways that could be utilized to understand and target HCC development from NASH. Knowing that HCC is associated with multiple etiologies, the emergence of NASH as a significant contributor makes the disease further divergent. Thus, future studies are warranted using a comprehensive cohort of patients from different ethnic groups.

## Figures and Tables

**Table 1:** Baseline clinical characteristics of NASH and NASH-HCC patients.

Characteristics	NASH	NASH-HCC	*p* Value
Age, median (25th–75th), (years)	62 (56.5–67.5)	63 (58–66)	
Gender distribution [n (%)]			
Male	24 (~77)	25 (~83)	
Female	7 (~23)	5 (~16)	
BMI, Median (25th–75th), kg/m^2^	30 (28–33.4)	28.5 (25.7–32)	
a TAG, Median (25th–75th), mg/dL	185 (143–231)	169 (155–189)	
b FBG, Median (25th–75th), mg/dL	98 (88–104)	106 (93–140)	
Insulin, Median (25th–75th), µU/mL	12 (10.2–13.6)	10.9 (7.8–13.1)	
c HOMA-IR, median (25th–75th)	3 (2.4–3.5)	2.9 (1.9–3.5)	
d AST, median (25th–75th), U/L	55 (44.5–65.5)	67 (59.7–67.7)	0.034
e ALT, median (25th–75th), U/L	37 (26–54.5)	45 (35.5–55.5)	
Albumin, median (25th–75th), g/dL	3.7 (3.4–3.9)	3.78 (2.9–3.8)	
Bilirubin, Median (25th–75th), mg/dL	1 (0.9–1.7)	1.1 (0.9–1.5)	
Hemoglobin, median (25th–75th), g/dL	11.3 (10.5–13.2)	12 (11.3–13.3)	
Platelets, median (25th–75th), 109/L	178 (115–212)	107 (62–194)	0.025
f TLC, median (25th–75th), 109/µL	6.5 (5.2–8)	6 (4.4–6)	0.041
Prothrombin, Median (25th–75th), %	88 (85–95)	70.9 (66.4–84)	0.001
g AFP, Median (25th–75th), ng/mL	9 (5.7–12)	312 (5.1–882)	0.033
h CEA, Mean ± SD, ng/mL	2.93 ± 0.1	2.94 ± 0.98	
i CA 19-9, Mean ± SD, U/mL	31.1 ± 3.6	43.2 ± 4.3	

a: TAG, triacylglycerol; b: FBG, fasting blood glucose; c: HOMA-IR, homeostatic model of insulin resistance; d: AST, aspartate aminotransferase; e: ALT, alanine aminotransferase; f: TLC, total leukocyte count; g: AFP, alfa fetoprotein; h: CEA, carcinoembryonic antigen; i: CA 19-9: cancer antigen; NASH: non-alcoholic steatohepatitis; HCC: hepatocellular carcinoma, n: number of samples.

**Table 2:** Non-targeted metabolites differed significantly in NASH-HCC as compared to NASH patients.

Metabolite	*p* Ajusted	Log 2 (Fold Change)	Regulation
Linoleic acid	0.096819	−2.06	Down
Indole-3-propionic acid	0.008281	−1.59	Down
(D/L)-leucine	0.001103	−1.46	Down
8(R)-Hydroperoxylinoleic acid	0.096819	0.61	Up
LysoPC 22:6/0:0	0.099088	0.91	Up
N-ethyl arachidonoyl amine	0.081234	1.38	Up
Cortisol	0.096819	1.38	Up
LysoPC O-15:1	0.026366	1.41	Up
13-HODE	0.052795	1.48	Up
9,10-DiHOME	0.004044	1.52	Up

**Table 3:** Targeted metabolites differed significantly in NASH-HCC as compared to NASH patients.

	Metabolite	*p* Value	Log 2 (Fold Change)	Regulation
**Targeted** **Non-quantified**	17:0 Lyso PE-d5	0.016402	−0.7244	Down
	19:0 Lyso PE-d5	0.014073	−0.67317	Down
	18:1 SM (d18:1/18:1)-d9	0.037147	−1.0716	Down
	20:1 SM (d18:1/20:1)-d9	1.92 × 10^−6^	−2.3629	Down
	22:1 SM (d18:1/22:1)-d9	1.41 × 10^−10^	−2.5234	Down
**Targeted quantified**	Ergocalciferol	0.018409	−1.1810	Down
	alpha-Tocopherol	0.015821	−0.9796	Down
	Folic acid	0.040606	−1.0113	Down
	Pantothenic acid	0.043206	−0.7311	Down
	Phylloquinone	0.006148	1.0917	Up

## Data Availability

The metabolomics generated data are available via “MassIVE” repository with study identifier [project ID: MSV000088003, https://massive.ucsd.edu/ProteoSAFe/dataset.jsp?task=25b6032dbf5f4973ab4c8f5600e6c4ba, ftp://massive.ucsd.edu/MSV000088003/] accessed on 25 November 2021.

## Supplementary Materials

The supporting information can be downloaded at: https://www.mdpi.com/article/10.3390/ijms24010210/s1.

## References

[1] Dufour J.-F., Scherer R., Balp M.-M., McKenna S.J., Janssens N., Lopez P., Pedrosa M. (2021). The global epidemiology of nonalcoholic steatohepatitis (NASH) and associated risk factors–A targeted literature review. Endocr. Metab. Sci..

[2] Kim G.-A., Lee H.C., Choe J., Kim M.-J., Lee M.J., Chang H.-S., Bae I.Y., Kim H.-K., An J., Shim J.H. (2018). Association between non-alcoholic fatty liver disease and cancer incidence rate. J. Hepatol..

[3] Cholankeril G., Patel R., Khurana S., Satapathy S.K. (2017). Hepatocellular carcinoma in non-alcoholic steatohepatitis: Current knowledge and implications for management. World J. Hepatol..

[4] Raza S., Rajak S., Anjum B., Sinha R.A. (2019). Molecular links between non-alcoholic fatty liver disease and hepatocellular carcinoma. Hepatoma Res..

[5] Kanwal F., Kramer J.R., Mapakshi S., Natarajan Y., Chayanupatkul M., Richardson P.A., Li L., Desiderio R., Thrift A.P., Asch S.M. (2018). Risk of hepatocellular cancer in patients with non-alcoholic fatty liver disease. Gastroenterology.

[6] Mittal S., El-Serag H.B., Sada Y.H., Kanwal F., Duan Z., Temple S., May S.B., Kramer J.R., Richardson P.A., Davila J.A. (2016). Hepatocellular carcinoma in the absence of cirrhosis in United States veterans is associated with nonalcoholic fatty liver disease. Clin. Gastroenterol. Hepatol..

[7] Paradis V., Zalinski S., Chelbi E., Guedj N., Degos F., Vilgrain V., Bedossa P., Belghiti J. (2009). Hepatocellular carcinomas in patients with metabolic syndrome often develop without significant liver fibrosis: A pathological analysis. Hepatology.

[8] Lewinska M., Santos-Laso A., Arretxe E., Alonso C., Zhuravleva E., Jimenez-Agüero R., Eizaguirre E., Pareja M.J., Romero-Gómez M., Arrese M. (2021). The altered serum lipidome and its diagnostic potential for Non-Alcoholic Fatty Liver (NAFL)-associated hepatocellular carcinoma. EBioMedicine.

[9] Khalil A., Elfert A., Ghanem S., Helal M., Abdelsattar S., Elgedawy G., Obada M., Abdel-Samiee M.A., El-Said H. (2021). The role of metabolomics in hepatocellular carcinomas. Egypt. Liver J..

[10] Pavlova N.N., Thompson C.B. (2016). The Emerging Hallmarks of Cancer Metabolism. Cell Metab..

[11] Negro F. (2020). Natural history of NASH and HCC. Liver Int..

[12] De Matteis S., Ragusa A., Marisi G., De Domenico S., Casadei Gardini A., Bonafe M., Giudetti A.M. (2018). Aberrant Metabolism in Hepatocellular Carcinoma Provides Diagnostic and Therapeutic Opportunities. Oxidative Med. Cell. Longev..

[13] Pan M.-X., Zheng C.-Y., Deng Y.-J., Tang K.-R., Nie H., Xie J.-Q., Liu D.-D., Tu G.-F., Yang Q.-H., Zhang Y.-P. (2021). Hepatic protective effects of Shenling Baizhu powder, a herbal compound, against inflammatory damage via TLR4/NLRP3 signalling pathway in rats with nonalcoholic fatty liver disease. J. Integr. Med..

[14] Wang M., Han J., Xing H., Zhang H., Li Z., Liang L., Li C., Dai S., Wu M., Shen F. (2016). Dysregulated fatty acid metabolism in hepatocellular carcinoma. Hepatic Oncol..

[15] Todisco S., Convertini P., Iacobazzi V., Infantino V. (2019). TCA Cycle Rewiring as Emerging Metabolic Signature of Hepatocellular Carcinoma. Cancers.

[16] Altman B.J., Stine Z.E., Dang C.V. (2016). From Krebs to clinic: Glutamine metabolism to cancer therapy. Nat. Rev. Cancer.

[17] Tenen D.G., Chai L., Tan J.L. (2020). Metabolic alterations and vulnerabilities in hepatocellular carcinoma. Gastroenterol. Rep..

[18] Léveillé M., Estall J.L. (2019). Mitochondrial Dysfunction in the Transition from NASH to HCC. Metabolites.

[19] Raza S., Tewari A., Rajak S., Sinha R.A. (2021). Vitamins and non-alcoholic fatty liver disease: A molecular insight. Liver Res..

[20] Sunshine H., Iruela-Arispe M.L. (2017). Membrane lipids and cell signaling. Curr. Opin. Lipidol..

[21] Younossi Z., Anstee Q.M., Marietti M., Hardy T., Henry L., Eslam M., George J., Bugianesi E. (2018). Global burden of NAFLD and NASH: Trends, predictions, risk factors and prevention. Nat. Rev. Gastroenterol. Hepatol..

[22] Clish C.B. (2015). Metabolomics: An emerging but powerful tool for precision medicine. Cold Spring Harb. Mol. Case Stud..

[23] Dhamija E., Paul S.B., Kedia S. (2019). Non-alcoholic fatty liver disease associated with hepatocellular carcinoma: An increasing concern. Indian J. Med. Res..

[24] Gawlik A., Shmoish M., Hartmann M.F., Wudy S.A., Olczak Z., Gruszczynska K., Hochberg Z. (2019). Steroid metabolomic signature of liver disease in nonsyndromic childhood obesity. Endocr. Connect..

[25] Wang K.K., Czaja A.J. (1988). Hepatocellular carcinoma in corticosteroid-treated severe autoimmune chronic active hepatitis. Hepatology.

[26] Tian Y., Li Y., Wang W.-X., Jiang W.-L., Fei J., Li C.-Y. (2020). Novel strategy for validating the existence and mechanism of the “gut–liver axis” in vivo by a hypoxia-sensitive NIR fluorescent probe. Anal. Chem..

[27] Albhaisi S.A., Bajaj J.S., Sanyal A.J. (2020). Role of gut microbiota in liver disease. Am. J. Physiol.-Gastrointest. Liver Physiol..

[28] Zhao Z.-H., Xin F.-Z., Xue Y., Hu Z., Han Y., Ma F., Zhou D., Liu X.-L., Cui A., Liu Z. (2019). Indole-3-propionic acid inhibits gut dysbiosis and endotoxin leakage to attenuate steatohepatitis in rats. Exp. Mol. Med..

[29] Wong R.J., Cheung R., Ahmed A. (2014). Nonalcoholic steatohepatitis is the most rapidly growing indication for liver transplantation in patients with hepatocellular carcinoma in the US. Hepatology.

[30] Liu F., Sun C., Chen Y., Du F., Yang Y., Wu G. (2021). Indole-3-Propionic Acid-Aggravated CCl4-Induced Liver Fibrosis via the TGF-β1/Smads Signaling Pathway. J. Clin. Transl. Hepatol..

[31] Han J., Dzierlenga A.L., Lu Z., Billheimer D.D., Torabzadeh E., Lake A.D., Li H., Novak P., Shipkova P., Aranibar N. (2017). Metabolomic profiling distinction of human nonalcoholic fatty liver disease progression from a common rat model. Obesity (Silver Spring Md.).

[32] Zhang B., Jiang M., Zhao J., Song Y., Du W., Shi J. (2022). The Mechanism Underlying the Influence of Indole-3-Propionic Acid: A Relevance to Metabolic Disorders. Front. Endocrinol..

[33] Zhang X., Coker O.O., Chu E.S., Fu K., Lau H.C., Wang Y.-X., Chan A.W., Wei H., Yang X., Sung J.J. (2021). Dietary cholesterol drives fatty liver-associated liver cancer by modulating gut microbiota and metabolites. Gut.

[34] Li Y., Xu W., Zhang F., Zhong S., Sun Y., Huo J., Zhu J., Wu C. (2020). The gut microbiota-produced indole-3-propionic acid confers the antihyperlipidemic effect of mulberry-derived 1-deoxynojirimycin. Msystems.

[35] Tajiri K., Shimizu Y. (2013). Branched-chain amino acids in liver diseases. World J. Gastroenterol..

[36] Sugiyama K., Yu L., Nagasue N. (1998). Direct effect of branched-chain amino acids on the growth and metabolism of cultured human hepatocellular carcinoma cells. Nutr. Cancer.

[37] Miuma S., Ichikawa T., Arima K., Takeshita S., Muraoka T., Matsuzaki T., Ootani M., Shibata H., Akiyama M., Ozawa E. (2012). Branched-chain amino acid deficiency stabilizes insulin-induced vascular endothelial growth factor mRNA in hepatocellular carcinoma cells. J. Cell. Biochem..

[38] Ninomiya S., Shimizu M., Imai K., Takai K., Shiraki M., Hara T., Tsurumi H., Ishizaki S., Moriwaki H. (2011). Possible role of visfatin in hepatoma progression and the effects of branched-chain amino acids on visfatin-induced proliferation in human hepatoma cells. Cancer Prev. Res..

[39] Hagiwara A., Nishiyama M., Ishizaki S. (2012). Branched-chain amino acids prevent insulin-induced hepatic tumor cell proliferation by inducing apoptosis through mTORC1 and mTORC2-dependent mechanisms. J. Cell. Physiol..

[40] Muto Y., Sato S., Watanabe A., Moriwaki H., Suzuki K., Kato A., Kato M., Nakamura T., Higuchi K., Nishiguchi S. (2006). Overweight and obesity increase the risk for liver cancer in patients with liver cirrhosis and long-term oral supplementation with branched-chain amino acid granules inhibits liver carcinogenesis in heavier patients with liver cirrhosis. Hepatol. Res..

[41] Kobayashi M., Ikeda K., Arase Y., Suzuki Y., Suzuki F., Akuta N., Hosaka T., Murashima N., Saitoh S., Someya T. (2008). Inhibitory effect of branched-chain amino acid granules on progression of compensated liver cirrhosis due to hepatitis C virus. J. Gastroenterol..

[42] Ichikawa K., Okabayashi T., Maeda H., Namikawa T., Iiyama T., Sugimoto T., Kobayashi M., Mimura T., Hanazaki K. (2013). Oral supplementation of branched-chain amino acids reduces early recurrence after hepatic resection in patients with hepatocellular carcinoma: A prospective study. Surg. Today.

[43] Behiry E.G., Mahmoud S.K., Swelim M.A., El-dougdoug K.A., Attia A., Hussein A.M. (2018). Changes in tryptophan and phenylalanine in chronic HCV patients treated with direct acting antiviral (sofosbuvir). Bull. Natl. Res. Cent..

[44] de Mello V.D., Sehgal R., Männistö V., Klåvus A., Nilsson E., Perfilyev A., Kaminska D., Miao Z., Pajukanta P., Ling C. (2021). Serum aromatic and branched-chain amino acids associated with NASH demonstrate divergent associations with serum lipids. Liver Int..

[45] Jain M., Nilsson R., Sharma S., Madhusudhan N., Kitami T., Souza A.L., Kafri R., Kirschner M.W., Clish C.B., Mootha V.K. (2012). Metabolite profiling identifies a key role for glycine in rapid cancer cell proliferation. Science.

[46] Gomes R.N., Felipe da Costa S., Colquhoun A. (2018). Eicosanoids and cancer. Clinics.

[47] Jee S.H., Kim M., Kim M., Yoo H.J., Kim H., Jung K.J., Hong S., Lee J.H. (2018). Metabolomics profiles of hepatocellular carcinoma in a Korean prospective cohort: The Korean Cancer Prevention Study-II. Cancer Prev. Res..

[48] Lu X., Yu H., Ma Q., Shen S., Das U.N. (2010). Linoleic acid suppresses colorectal cancer cell growth by inducing oxidant stress and mitochondrial dysfunction. Lipids Health Dis..

[49] Ma C., Kesarwala A.H., Eggert T., Medina-Echeverz J., Kleiner D.E., Jin P., Stroncek D.F., Terabe M., Kapoor V., ElGindi M. (2016). NAFLD causes selective CD4+ T lymphocyte loss and promotes hepatocarcinogenesis. Nature.

[50] Paul B., Lewinska M., Andersen J.B. (2022). Lipid alterations in chronic liver disease and liver cancer. JHEP Rep..

[51] Van Meer G., Voelker D.R., Feigenson G.W. (2008). Membrane lipids: Where they are and how they behave. Nat. Rev. Mol. Cell Biol..

[52] Zheng J.-S., Xu A., Huang T., Yu X., Li D. (2012). Low docosahexaenoic acid content in plasma phospholipids is associated with increased non-alcoholic fatty liver disease in China. Lipids.

[53] Tiwari-Heckler S., Gan-Schreier H., Stremmel W., Chamulitrat W., Pathil A. (2018). Circulating phospholipid patterns in NAFLD patients associated with a combination of metabolic risk factors. Nutrients.

[54] Gorden D.L., Myers D.S., Ivanova P.T., Fahy E., Maurya M.R., Gupta S., Min J., Spann N.J., McDonald J.G., Kelly S.L. (2015). Biomarkers of NAFLD progression: A lipidomics approach to an epidemic1 [S]. J. Lipid Res..

[55] Skill N.J., Scott R.E., Wu J., Maluccio M.A. (2011). Hepatocellular carcinoma associated lipid metabolism reprogramming. J. Surg. Res..

[56] Puri P., Baillie R.A., Wiest M.M., Mirshahi F., Choudhury J., Cheung O., Sargeant C., Contos M.J., Sanyal A.J. (2007). A lipidomic analysis of nonalcoholic fatty liver disease. Hepatology.

[57] Rein-Fischboeck L., Haberl E.M., Pohl R., Feder S., Liebisch G., Krautbauer S., Buechler C. (2019). Variations in hepatic lipid species of age-matched male mice fed a methionine-choline-deficient diet and housed in different animal facilities. Lipids Health Dis..

[58] Musso G., Cassader M., Paschetta E., Gambino R. (2018). Bioactive lipid species and metabolic pathways in progression and resolution of nonalcoholic steatohepatitis. Gastroenterology.

[59] Li Y., Lin N., Xu J., Lu Y., Chen S., Pan C., Wang C., Xu M., Zhou B., Xu R. (2018). Measurement of serum and hepatic eicosanoids by liquid chromatography tandem-mass spectrometry (LC-MS/MS) in a mouse model of hepatocellular carcinoma (HCC) with delivery of c-Met and activated β-catenin by hepatocyte hydrodynamic injection. Med. Sci. Monit. Int. Med. J. Exp. Clin. Res..

[60] Hardwick J.P., Osei-Hyiaman D., Wiland H., Abdelmegeed M.A., Song B.J. (2009). PPAR/RXR Regulation of Fatty Acid Metabolism and Fatty Acid omega-Hydroxylase (CYP4) Isozymes: Implications for Prevention of Lipotoxicity in Fatty Liver Disease. PPAR Res..

[61] de la Cruz-Ojeda P., Flores-Campos R., Dios-Barbeito S., Navarro-Villarán E., Muntané J. (2021). Role of Nitric Oxide in Gene Expression Regulation during Cancer: Epigenetic Modifications and Non-Coding RNAs. Int. J. Mol. Sci..

[62] Zhang Q., Zhang Y., Hu X., Qin Y., Zhong W., Meng J., Xiao T., Zhang C., Li M., Chen S. (2017). Thymidine phosphorylase promotes metastasis and serves as a marker of poor prognosis in hepatocellular carcinoma. Lab. Investig..

[63] Da Li F.-F.B., Chen N.-N., Cao J.-M., Sun W.-P., Zhou Y.-M., Cao C., Li C.-Y., Yang Q. (2014). Epigenetic repression of phosphatidylethanolamine N-methyltransferase (PEMT) in BRCA1-mutated breast cancer. Oncotarget.

[64] European Association For The Study Of The Liver (2018). EASL clinical practice guidelines: Management of hepatocellular carcinoma. J. Hepatol..

[65] Kleiner D.E., Brunt E.M., Van Natta M., Behling C., Contos M.J., Cummings O.W., Ferrell L.D., Liu Y.-C., Torbenson M.S., Unalp-Arida A. (2005). Design and validation of a histological scoring system for nonalcoholic fatty liver disease. Hepatology.

[66] Kleiner D., Makhlouf H., Program D., Investigation P., Branch R., Shams A. (2017). Histology of NAFLD and NASH in adults and children. Clin. Liver Dis..

[67] Group F.M.C.S., Bedossa P. (1994). Intraobserver and interobserver variations in liver biopsy interpretation in patients with chronic hepatitis C. Hepatology.

[68] Altman D.G., McShane L.M., Sauerbrei W., Taube S.E. (2012). Reporting recommendations for tumor marker prognostic studies (REMARK): Explanation and elaboration. BMC Med..

[69] Huang X.-J., Choi Y.-K., Im H.-S., Yarimaga O., Yoon E., Kim H.-S. (2006). Aspartate aminotransferase (AST/GOT) and alanine aminotransferase (ALT/GPT) detection techniques. Sensors.

[70] Pagana K.D., Pagana T.J., Pagana T.N. (2022). Mosby’s^®^ Diagnostic and Laboratory Test Reference-E-Book.

[71] Verrijken A., Francque S., Mertens I., Prawitt J., Caron S., Hubens G., Van Marck E., Staels B., Michielsen P., Van Gaal L. (2014). Prothrombotic factors in histologically proven nonalcoholic fatty liver disease and nonalcoholic steatohepatitis. Hepatology.

[72] Matthews D.R., Hosker J.P., Rudenski A.S., Naylor B.A., Treacher D.F., Turner R.C. (1985). Homeostasis model assessment: Insulin resistance and beta-cell function from fasting plasma glucose and insulin concentrations in man. Diabetologia.

[73] Cajka T., Fiehn O. (2016). Toward merging untargeted and targeted methods in mass spectrometry-based metabolomics and lipidomics. Anal. Chem..

[74] Lin C.Y., Wu H., Tjeerdema R.S., Viant M.R. (2007). Evaluation of metabolite extraction strategies from tissue samples using NMR metabolomics. Metabolomics.

[75] Aldana J., Romero-Otero A., Cala M.P. (2020). Exploring the lipidome: Current lipid extraction techniques for mass spectrometry analysis. Metabolites.

[76] Menegollo M., Tessari I., Bubacco L., Szabadkai G. (2019). Determination of ATP, ADP, and AMP levels by reversed-phase high-performance liquid chromatography in cultured cells. Calcium Signalling.

[77] Bell E.C., John M., Hughes R.J., Pham T. (2014). Ultra-performance liquid chromatographic determination of tocopherols and retinol in human plasma. J. Chromatogr. Sci..

[78] Zhang Y., Bilbao A., Bruderer T., Luban J., Strambio-De-Castillia C., Lisacek F.d.r., Hopfgartner G.r., Varesio E. (2015). The use of variable Q1 isolation windows improves selectivity in LC–SWATH–MS acquisition. J. Proteome Res..

[79] Bonner R., Hopfgartner G. (2016). SWATH acquisition mode for drug metabolism and metabolomics investigations. Bioanalysis.

[80] Tsugawa H., Cajka T., Kind T., Ma Y., Higgins B., Ikeda K., Kanazawa M., VanderGheynst J., Fiehn O., Arita M. (2015). MS-DIAL: Data-independent MS/MS deconvolution for comprehensive metabolome analysis. Nat. Methods.

[81] Overmyer K.A., Shishkova E., Miller I.J., Balnis J., Bernstein M.N., Peters-Clarke T.M., Meyer J.G., Quan Q., Muehlbauer L.K., Trujillo E.A. (2021). Large-scale multi-omic analysis of COVID-19 severity. Cell Syst..

[82] Gromski P.S., Xu Y., Kotze H.L., Correa E., Ellis D.I., Armitage E.G., Turner M.L., Goodacre R. (2014). Influence of missing values substitutes on multivariate analysis of metabolomics data. Metabolites.

[83] Li B., Tang J., Yang Q., Cui X., Li S., Chen S., Cao Q., Xue W., Chen N., Zhu F. (2016). Performance evaluation and online realization of data-driven normalization methods used in LC/MS based untargeted metabolomics analysis. Sci. Rep..

[84] Huang J., Mondul A.M., Weinstein S.J., Koutros S., Derkach A., Karoly E., Sampson J.N., Moore S.C., Berndt S.I., Albanes D. (2016). Serum metabolomic profiling of prostate cancer risk in the prostate, lung, colorectal, and ovarian cancer screening trial. Br. J. Cancer.

[85] Aredo J.V., Purington N., Su L., Luo S.J., Diao N., Christiani D.C., Wakelee H.A., Han S.S. (2021). Metabolomic profiling for second primary lung cancer: A pilot case-control study. Lung Cancer.

[86] Henglin M., Claggett B.L., Antonelli J., Alotaibi M., Magalang G.A., Watrous J.D., Lagerborg K.A., Ovsak G., Musso G., Demler O.V. (2022). Quantitative Comparison of Statistical Methods for Analyzing Human Metabolomics Data. Metabolites.

[87] Chong J., Xia J. (2018). MetaboAnalystR: An R package for flexible and reproducible analysis of metabolomics data. Bioinformatics.

[88] Cavill R., Kamburov A., Ellis J.K., Athersuch T.J., Blagrove M.S., Herwig R., Ebbels T.M., Keun H.C. (2011). Consensus-phenotype integration of transcriptomic and metabolomic data implies a role for metabolism in the chemosensitivity of tumour cells. PLoS Comput. Biol..

