# Supplementary figures and images for: Metabolomics and Lipidomics Screening Reveal Reprogrammed Signaling Pathways toward Cancer Development in Non-Alcoholic Steatohepatitis
Source: Int J Mol Sci. 2022 Dec 22;24(1):210. doi: 10.3390/ijms24010210 (PMC9820351; doi:10.3390/ijms24010210)

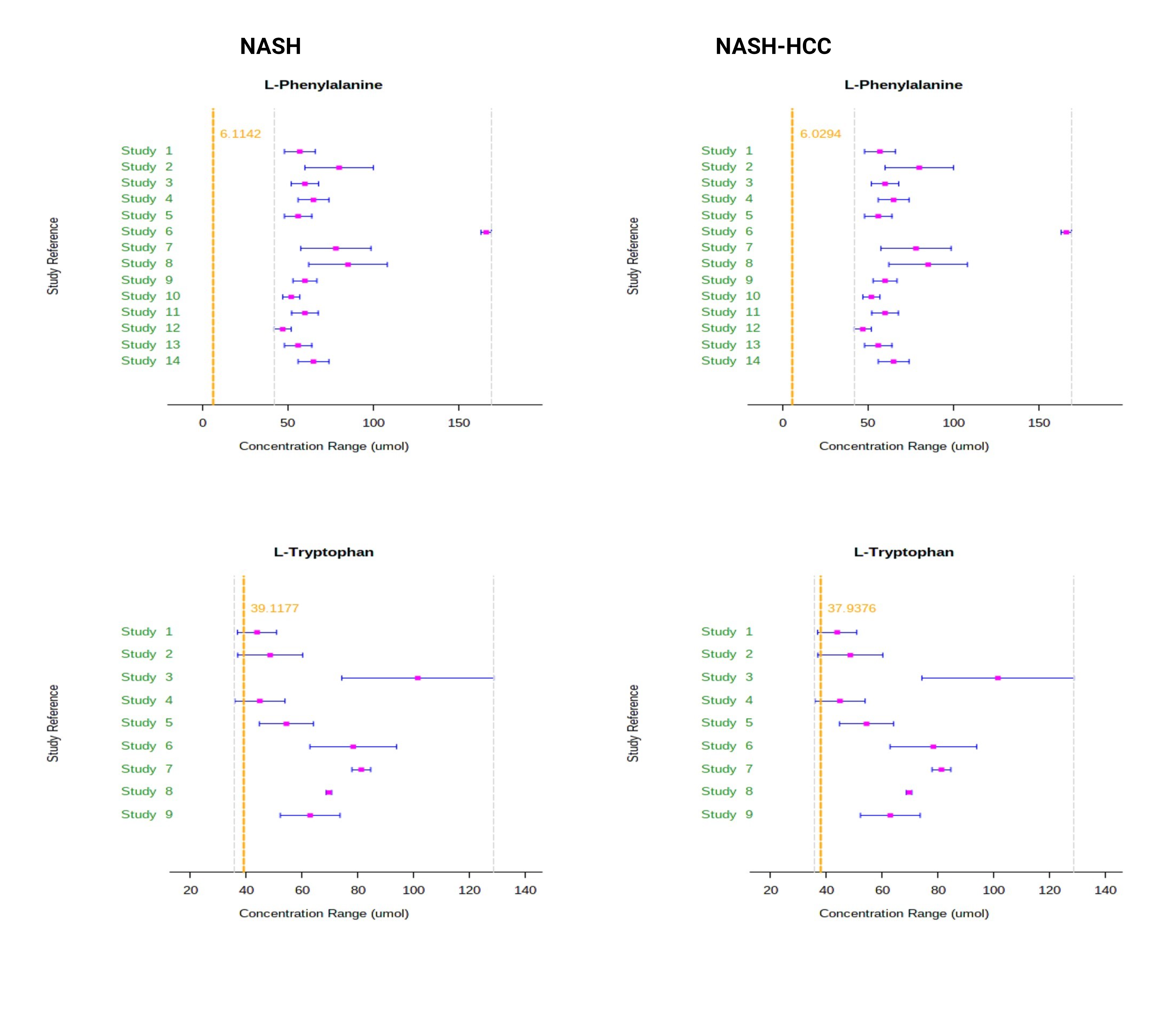

Supplement: Supplementary file 1 [file ijms-24-00210-s001.zip › ijms-1993632-supplementary/Figure S1-A (Amino acids.png]

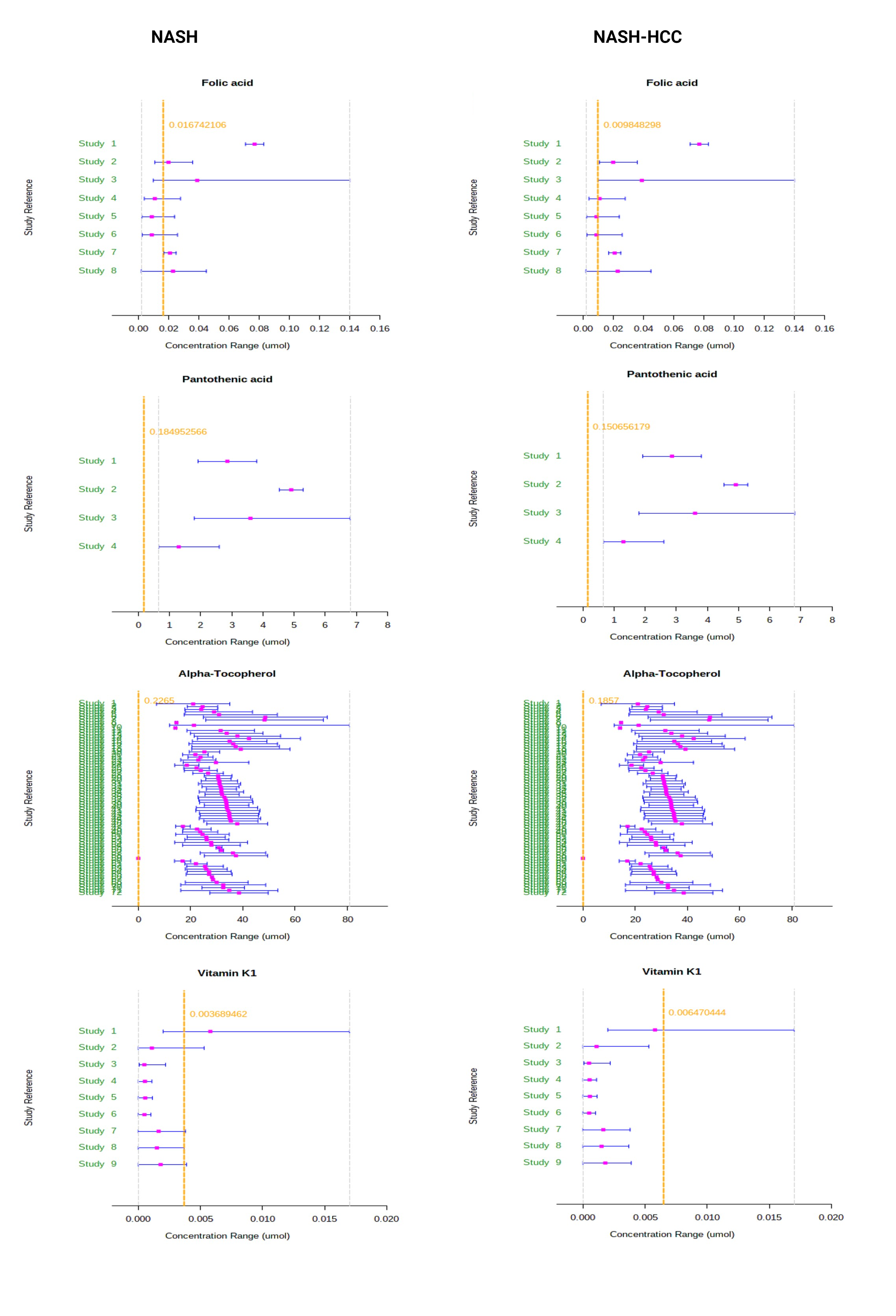

Supplement: Supplementary file 1 [file ijms-24-00210-s001.zip › ijms-1993632-supplementary/Figure S1-B (Vitamins) .png]

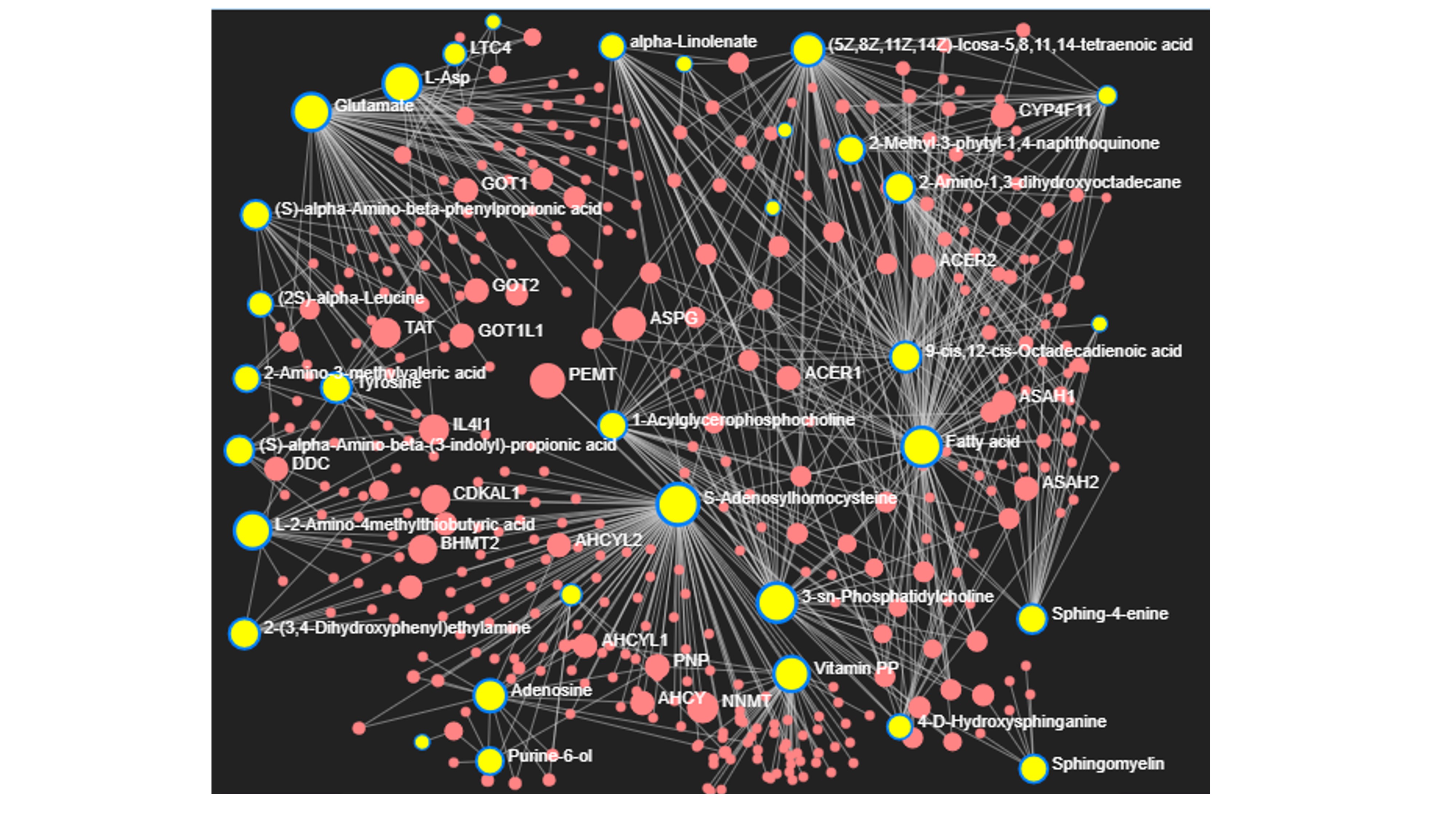

Supplement: Supplementary file 1 [file ijms-24-00210-s001.zip › ijms-1993632-supplementary/Figure S2 (metabolites-proteins interaction).jpg]
